# Comparison of Immune Response between SARS, MERS, and COVID-19 Infection, Perspective on Vaccine Design and Development

**DOI:** 10.1155/2021/8870425

**Published:** 2021-01-22

**Authors:** Hossein Ansariniya, Seyed Mohammad Seifati, Erfan Zaker, Fateme Zare

**Affiliations:** ^1^Department of Immunology, School of Medicine, Shahid Beheshti University of Medical Sciences, Tehran, Iran; ^2^Reproductive Immunology Research Center, Shahid Sadoughi University of Medical Sciences, Yazd, Iran; ^3^Department of Medical Genetics, Faculty of Medicine, Shahid Sadoughi University of Medical Sciences, Yazd, Iran; ^4^Department of Immunology, School of Public Health, Tehran University of Medical Sciences, Tehran, Iran

## Abstract

Severe Acute Respiratory Syndrome (SARS), Middle East Respiratory Syndrome (MERS), and Coronavirus Disease 2019 (COVID-19) infections are the three epidemiological diseases caused by the Coronaviridae family. Perceiving the immune responses in these infections and the escape of viruses could help us design drugs and vaccines for confronting these infections. This review investigates the innate and adaptive immune responses reported in the infections of the three coronaviruses SARS, MERS, and COVID-19. Moreover, the present study can trigger researchers to design and develop new vaccines and drugs based on immune system responses. In conclusion, due to the need for an effective and efficient immune stimulation against coronavirus, a combination of several strategies seems necessary for developing the vaccine.

## 1. Introduction

**Figure 1:**
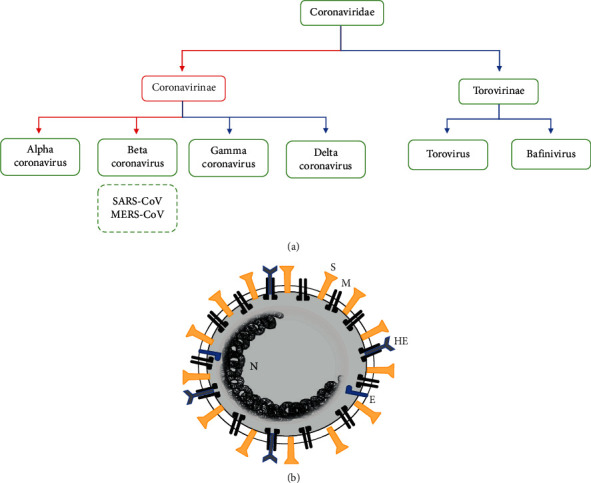
(a) Classification of the Coronaviridae family. Alpha coronaviruses and beta coronaviruses primarily cause respiratory and intestinal infection in mammals, while gamma coronaviruses and delta coronaviruses mainly infect birds. (b) Model of coronavirus structure; structural proteins in coronavirus are the spike (S), membrane (M), envelope (e) glycoproteins, hemagglutinin esterase (HE), and nucleocapsid (N) protein.

The Coronaviridae is recognized as a novel virus family of enveloped and single-strand RNA (ssRNA) viruses [1]. The Coronaviridae includes two subfamilies, the Coronavirinae and the Torovirinae. Coronaviridae causes mild respiratory and gastrointestinal infections in mammals and birds as indicated by the molecular and serological aspects of Coronaviridae. More specifically, this classification is shown in Figure 1(a). In recent years, Coronaviridae family members have received much attention because these viruses have been the main cause of several epidemiological outbreaks in worldwide [2]. In 2002, a contagious infectious disease, known as Severe Acute Respiratory Syndrome (SARS) infection, showed to be associated with coronaviruses [3]. The genome sequence showed that SARS-associated Coronavirus (SARS-CoV) is a new virus and has no genetic connection with any known human coronaviruses [4]. SARS-CoV is believed to be an animal virus from the animal reservoir, possibly bats, which diffuse to different animals (civet cats). Contaminated people by SARS-CoV were first reported in the Guangdong Province of China in 2002 [5]. The Middle East Respiratory Syndrome (MERS) is a viral respiratory illness brought about by a new coronavirus (MERS-CoV) that was first recognized in Saudi Arabia in 2012 [6]. On 31 December 2019, pneumonia caused by an obscure reason in Wuhan, China, was first announced to the World Health Organization (WHO) [4]. Later, the disease was called coronavirus disease 2019 (COVID-19) or Severe Acute Respiratory Syndrome Coronavirus 2 (SARS-CoV-2), which has spread to most of the world countries, showing a mortality rate of approximately 3.7% compared with a mortality rate of under 0.1% from influenza (https://www.who.int). Similar to SARS-CoV and MERS-CoV, the SARS-CoV-2 is also caused by the beta-coronavirus family [7]. There seems to be a critical requirement for effective treatment. The running focus is on the expansion of new therapeutics, including antivirals and vaccines.

Since the immune system has a protective role in most infections, knowledge about the immune response against coronaviruses can help develop drugs and vaccines for these diseases. The present study investigates innate and adaptive immune responses reported in the infections of the three coronaviruses SARS, MERS, and COVID-19. Collectively, the findings of this study can help researchers to design and develop new vaccines and drugs based on immune system responses.

## 2. The Function of the Most Important SARS, MERS, and COVID-19 Encode Proteins

Four major structural proteins including spike (S), membrane (M), envelope (E), and nucleocapsid (N) proteins are encoded by the coronavirus [8]. The S glycoprotein binds to the receptors angiotensin-converting enzyme 2 (ACE2) for SARS-CoV and dipeptidyl peptidase 4 (DPP4, also known as CD26) for MERS-CoV on the surface of host cells and induces fusion of the viral envelope with cell membranes and facilitates attachment [9]. It is worth mentioning that, among the fourteen proteins identified in SARS-CoV, eight of them including the replicase, S protein, open reading frame (10)3, Orf4, E protein, M protein, Orf13, and N proteins are recognized to be able to stimulate T cells. But, the highest response is produced by structural proteins such as S, E, M, and N [10]. Replicase and Orfs are the nonstructural proteins in SARS-CoV.

One of the reasons for the rapid spread of COVID-19 relative to SARS and MERS-CoV could be a structural difference in the S protein which allows the virus to escape from the host immune system [11]. Besides, the S protein can release interleukin- (IL-) 8 through activating mitogen-activated protein kinase (MAPK) and activator protein 1 (AP-1) [11].

M protein can be considered as the most abundant protein in the virion. This protein in combination with the nucleocapsid plays an essential role in an organized assembly of the particles [12]. The small E glycoprotein is essential for virus budding. This glycoprotein plays a crucial role in the accumulation and morphogenesis of virions within the cell [13].

The N protein of the coronavirus is a part of the nucleocapsid which is involved in replication and transcription of the genome [14]. Additionally, this protein is also able to activate the AP-1 pathway. More specifically, the expression of this protein in African green monkey cell line and human hepatocellular carcinoma cell line can enhance the number of transcription factors binding to the promoter sequence of c-Fos and activate the transcription factor 2 (ATF2), cAMP-responsive element-binding protein 1 (CREB1), and FosB. As a result, the activation of the important cellular pathways seems to be selective by this protein [15]. Kopecky-Bromberg et al. [16] indicated that the SARS-CoV, Orf3b, Orf6, and N proteins act as interferon antagonists through different mechanisms. The N proteins use phosphorylation of IFN regulatory factor 3 (IRF3) and binding of IRF-3 as a promoter with IRF-3 binding sites to inhibit the interferon beta (IFN-*β*) and IRF-3 expression. However, the N protein inhibits interferon synthesis; it does not inhibit interferon signaling.

The envelopes of some coronaviruses such as human coronavirus OC43 (HCoV-OC43) and human coronavirus HKU1 (HCoV-HKU1) also possess a hemagglutinin-esterase glycoprotein (HE) which makes short spikes [17]. This protein does not appear to be present in the structure of SARS and MERS-CoV. A model of coronavirus structure is shown in Figure 1(b).

## 3. Immunopathology of Coronavirus

For most patients, COVID-19 may only affect the lungs since it is primarily a respiratory illness [7]. The complications of COVID-19 seem to be lower than MERS and SARS. The most common symptoms of COVID-19, like clinical features of SARS and MERS, are fever, fatigue, and respiratory symptoms, including sore throat, cough, and shortness of breath. While diarrhea occurs in about 20-25% of SARS and MERS patients, intestinal problems have been added to the list of symptoms of patients with COVID-19 at the Centers for Disease Control and Prevention (CDC) recently. Furthermore, most sufferers have developed pneumonia with typical pulmonary ground-glass opacity adjustments in chest CT and lymphopenia [18–20]. Besides, hospitalized patients with severe COVID-19 indicated high levels of cytokines including IL-10, IL-7, IL-2, granulocyte colony-stimulating factor (G-CSF), C-X-C motif chemokine 10/interferon gamma-induced protein 10 (CXCL10/IP-10), monocyte chemoattractant protein-1 (MCP-1), macrophage inflammatory protein 1 alpha (MIP-1A), and tumor necrosis factor-alpha (TNF-*α*) [19]. These findings are in line with SARS and MERS in that the presence of lymphopenia and “cytokine storm” may play a significant role in the pathogenesis of COVID-19 [21–23].

Once the virus enters the respiratory epithelial cells, SARS-CoV-2 elicits an immune response with poor production of IFN. Proinflammatory immune responses are mediated by Fc-gamma-Receptor IIA (Fc*γ*RIIA), nuclear factor-kappa B (NF-*κ*B) p65, and p38 MAPK by T helper 1 (Th1) cells and intermediate CD14^+^ CD16^+^ monocytes. As a consequence, macrophages and neutrophils infiltrate into the lung tissue which results in a cytokine storm [24]. Furthermore, granulocyte macrophage-colony stimulating factor (GM-CSF) causes the starting activity of CD14^+^ CD16^+^ inflammatory monocytes to produce large quantities of IL-6 and TNF-*α* [25]. Cytokine storm may be associated with the severity of the disease and trigger the pathological processes prompting disseminated intravascular coagulation (DIC), intravascular permeability, plasma leakage, and representing dangerous respiratory manifestations [26]. Most cases of COVID-19 (approximately 80%) are asymptomatic or have slight symptoms; however, in about fifteen percent of instances, cytokine storm can lead to lung harm and viral sepsis caused by inflammation, which leads to different problems such as acute respiratory distress syndrome [27], respiratory failure, shock, pneumonitis, organ failure, and even death [7].

## 4. Innate Immune Response in SARS and MERS Infection

Neutrophils are considered as the first line of the innate immune response. Studies on MERS-CoV infection revealed that neutrophil chemoattractant chemokine IL-8 (CXCL8) is highly expressed in the lower respiratory tract of the patient [28]. IL-8 plays an essential role in recruitment, activation, and accumulation of neutrophil in the site of infection and subsequently induce the formation of neutrophil extracellular traps (NETs). NETs directly cause inflammation and increase the secretion of IL-8, resulting in the further recruitment of neutrophils to the site of infection [29]. Dendritic cells (DC) and macrophages are the other components in the innate immune network. Plasmacytoid DC is activated with viruses or their derivatives and produces large amounts of IFN type I. IFN-I induces direct antiviral responses in the infected cell [30]. Macrophages also produce IFN-I and other proinflammatory cytokines which triggers both a protective response against the virus and potentially pathological complications of the disease [31]. Tseng et al. identified the effect of SARS-CoV infection on human macrophages and DCs. Their study demonstrated that DCs and macrophages are not effective against infection in SARS-CoV and phenotypically altered during this infection [32].

Speigel et al. showed that SARS-CoV is a weak inducer of IFN-I in the infected cell. They reported that when immature DC is activated with SARS, it causes a defect in upregulation of major histocompatibility complex class-I (MHC-I) in these cells, and the function of DC was impaired [33].

High serum levels of proinflammatory cytokines were observed in MERS-CoV and SARS-CoV infection, indicating a potential similar cytokine storm-mediated illness severity [22, 23]. In fact, several studies have been performed on cytokines secreted in SARS-CoV infection, and the result has shown that IP-10, IL-1, TNF-*α*, IL-6, IL-8, and MCP-1 were increased in the blood of the patients infected with SARS-CoV [34].

**Figure 2:**
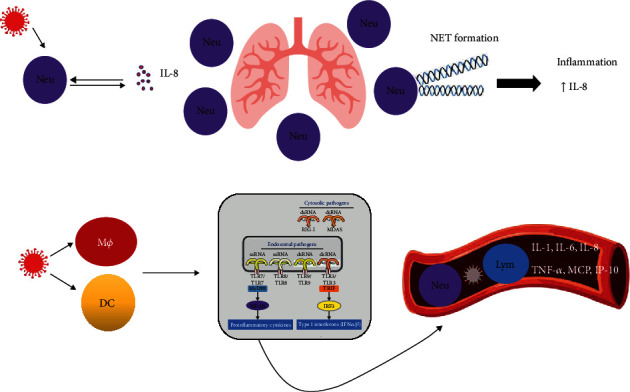
Schematic diagram of innate immune responses in SARS-CoV and MERS infection. Neutrophils secreted chemoattractant chemokine interleukin- (IL-) 8. IL-8 is an essential role in recruitment, activation, and accumulation of neutrophil in the site of infection and subsequently induces the formation of neutrophil extracellular traps (NETs). NETs directly cause the inflammation and increase the secretion of IL-8. Dendritic cells (DC) and macrophages (Mф) activated with viruses or their derivatives and produce large amounts of IFN type I and proinflammatory cytokine such as IL-1, IL-6, IL-8, tumor necrosis factor-alpha (TNF-*α*), monocyte chemoattractant protein-1 (MCP), and interferon gamma-induced protein 10.

For arranging an antiviral reaction, innate immune cells need to detect the virus via pathogen-recognition receptors (PRRs). Both endosomal (Toll-like receptor- (TLR-) 3 and TLR-7) and cytosolic (retinoic acid-inducible gene I (RIG-I)/melanoma differentiation-associated protein (MDA5)) receptors are involved. For RNA viruses like coronavirus, the pathogen-associated molecular patterns (PAMPs) in the structure of viral genomic RNA or within viral replication, including double-strand RNA (dsRNA), are identified by PRRs. This activates the downstream signaling cascade, i.e., NF-*κ*B and IRF3. In the nuclei, these transcription factors instigate the expression of IFN-I and other proinflammatory cytokines. This primary response includes the first-line protection towards viral contamination at the entry site [35]. The schematic demonstration of the innate immune response against Coronaviruses is illustrated in Figure 2.

Faure et al. in their study explored that the expression of RIG-1, MDA-5, and IRF3 in MERS-CoV-infected patients with poor outcomes was decreased which led to a considerable reduction in the IFN-*α* expression. Also, this research demonstrated the association of poor outcome in MERS-CoV infection with uncontrolled and persistence of IL-10 and CXCL10 secretion [36]. In an in vitro microarray analysis, Josset et al. reported that MERS-CoV could induce the IL-17A expression compared with SARS-CoV [37].

## 5. Innate Immune Response in SARS-CoV-2 Infection

Varying degrees of immune interference is expected in SARS-CoV-2 infection. Interestingly, virus transmission is documented even among asymptomatically infected people. This may indicate a postponed early reaction in the innate immune response. Moreover, no severe instances were mentioned in young children when the innate immune response was significant. This information strongly suggests that the innate immune response is a critical element in the disease's outcome [7].

Currently, limited data is available on the host innate immune status of patients infected with SARS-CoV-2. The development of strong innate immunity at the beginning of the infection with viruses may prevent the viral spread and hyperimmune activation. RNA profiling of bronchoalveolar lavage (BAL) [38] cells depicted that the expression of the proinflammatory chemokines and cytokine genes was markedly increased in patients infected with SARS-CoV. Unlike SARS-CoV, which produces an inadequate interferon response, SARS-CoV-2 stimulates the overexpression of IFN-induced genes (ISGs). These ISGs increase expression of inflammatory genes and exhibit immunopathological activity [39].

Farshi et al. attempted to emphasize the effective role of phagocytes in the elimination of SARS-CoV-2 in both the mouse model and the human model. More specifically, the phagocytic cells including monocyte-derived infiltrating macrophages and alveolar macrophages along with antibodies play a considerable role in the control of infection. In addition, they observed natural antibodies were probably in the early stages of infection. They also evaluated the level of IgM and IgG in children and adults which led to the existence of a high level of IgM and IgG in children compared to adult patients [40]. It must be noted that these antibodies, which are mainly produced against blood type antigens, may contribute to decreasing the viral load along with the activation of complement through the classical pathway [41].

According to the data obtained from the mouse model, depleted from natural killer (NK) cells, the virus was eradicated in these mice. Consequently, antibody-dependent cellular cytotoxicity (ADCC) mediated by NK cells was not a mechanism to fight the SARS-CoV-2 [40]. It is quite evident that phagocytes, natural antibodies, complement, and cytokine play a key role in innate immunity against SARS-CoV-2 infection.

## 6. Adaptive Immune Response in SARS and MERS Infection

**Figure 3:**
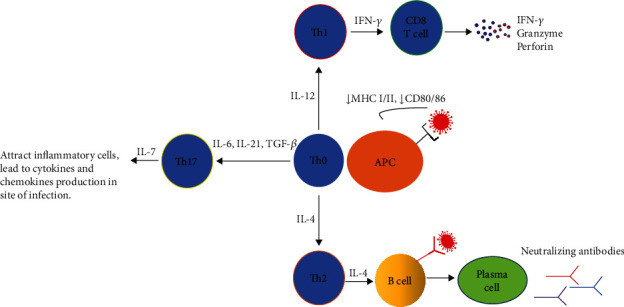
Adaptive immune response to coronaviruses infection. Dendritic cells presented the viral antigens to T lymphocytes. T cells differentiate into different subtypes under the influence of secreted cytokines. Th1 helps CD8^+^ T cells to eradicate infected cells by secreting interferon-gamma. Th17 cytokines attract inflammatory cells to the infection site, and IL-4 produced by Th2 cells activates B cells to secrete neutralizing antibodies.

As is presented in Figure 3, a simple schematic diagram of an adaptive immune response in SARS and MERS infection can be illustrated. Regarding the literature, one investigation mentioned that CD8^+^ T cell responses were more extensive than CD4^+^ T cell responses in SARS-CoV infection [42]. Another study demonstrated that primary infection with SARS-CoV resulted in a severe reduction in MERS-CoV titers due to cross-reaction five days after infection. As a result, the cross-reactive T cell response may lead to a downward trend in MERS-CoV titer [43]. Besides, during MERS-CoV infection, the virus attacks the immune system and downregulates MHC-I, MHC-II, and CD80/86 in antigen-presenting cells (APC), which in consequence inhibits T cell response [44]. Recently, the induction of immune system suppression during MERS-CoV infection employing promotion T cell apoptosis has been recognized as another way to manipulate the host immune responses for surviving the virus [45]. During MERS-CoV infection, the number of CD8^+^ T cells is in line with the severity of the disease. In other words, patients with severe/moderate infection reveal high frequencies of CD8^+^ T cells whereas CD4^+^ T cell and antibody response are minimally distinguished in this phase [46]. Both CD4^+^ and CD8^+^ T cells isolated from human peripheral blood and lymphatic organs can be contaminated with MERS-CoV but not with SARS-CoV. This infection pattern may be attributed to the low expression of the ACE2-SARS-CoV receptor in T cells [47]. There is a significant upregulation level of the IL-17 expression in patients infected with MERS-CoV [22, 23]. T helper cells, particularly Th17 cells, produce IL-17 proinflammatory cytokines through signal transducers and activators of transcription 3 (STAT3) and NF-*κ*B signaling pathways [48]. This information proposes that MERS-CoV contamination induces Th17 cytokines. These Th17 cytokines can attract inflammatory cells to the infection site, leading to the activation of different cytokine and chemokine downstream cascades, such as IL-1, IL-6, TNF-*α*, transforming growth factor-beta (TGF-*β*), IL-8, and MCP-1 [49]. In a study, mice vaccinated with SARS-CoVs modified glycoprotein DNA, expanded protective immunity from T cell induction, and manufactured neutralizing antibodies. This protective immunity was mainly due to the antibody-dependent (instead of T cell-dependent) response [50]. There was a significant association of strong T cell response with higher neutralizing antibodies, while more serum Th2 cytokines (IL-4, IL-5, IL-10) were observed in the fatal group [42]. The delayed and inadequate response to coronavirus infection is related to severe consequences for both types of coronavirus infection [51]. The MERS-CoV antibody response is detected on days 14-21 after infection. Antibody concentrations elevate over time and remain for more than 18 months, and long-term antibody response relies on the severity of the infection [52–54]. The SARS-CoV antibody response can be detected up to two years after infection and then gradually reduced until it completely disappears after 6 years of contamination [51, 55]. Coronaviruses have been shown to express surface spike proteins which are the dominant antigenic proteins and stimulate the response of antibodies. In one study, it was reported that mice immunized with coronavirus S nanoparticles produce high degrees of neutralizing antibodies toward homologous viruses [56]. By using recombinant MERS-CoV spike protein subunit 1-relying enzyme-linked immunosorbent assay (ELISA), an antibody against S protein was found to be desirable for screening [57]. The existence of long-term antibodies in most patients may be clarified by MERS-CoV contamination inciting memory B cells. As a result, these antibodies may protect people from MERS-CoV reinfection [58].

## 7. Adaptive Response in SARS-CoV-2 Infection

Adaptive immunity in SARS-CoV-2 infection involves T and B cell immunity and antiviral neutralizing antibody response. Virus-infected respiratory epithelial cells present viral peptides to CD8^+^ cytotoxic T cells via MHC-I. CD8^+^ T cells kill virus-infected cells utilizing perforin and granzyme [59]. Viral particles are presented to CD4^+^ T cells by professional APC which are often DC and macrophage cells via MHC-II [60]. CD147 which is expressed in many cells and tissues is also involved in apoptosis, cell differentiation, proliferation, and tumor metastasis [27]. Accordingly, block CD147 with an antibody against it (meplazumab) may act a crucial role in the treatment of patients with COVID-19 [61]. A reduction in the number of lymphocytes including Th, cytotoxic T lymphocytes (CTL), and B cells was observed during the disease [62]. In COVID-19 patients, a descending trend in lymphocytes is regarded as the disease progresses [63]. One possible explanation for the cause of lymphopenia in COVID-19 patients is the exhaustion in CD8^+^ T cells or induction of apoptosis [59]. Lymphopenia is considered as a major criterion for this infection and is proportional to the severity of the disease. Other viral agents, such as the human immunodeficiency virus (HIV), can lead to lymphopenia as well [64]. Various drugs are effective in lymphocyte proliferation or inhibit apoptosis including IL-2 and IL-7, to name just a few, can assist in preventing lymphopenia and restoring lymphocytes in severe cases [63]. It is clear that the percentage of lymphocytes is regarded as a predictive marker indicating the severity or recovery of the disease [65].

It is interesting to note that B cells can directly detect the SARS-CoV-2 and interact with CD4^+^ T cells. In most of the infected individuals, IgM and IgG antibody serum levels can be spotted 1-2 weeks after the onset of the symptoms [66]. Although no clear association has been reported between humoral and cellular immune responses regarding disease severity and symptoms, high levels of anti-SARS-CoV-2 antibodies have been reported in convalescence patients [67]. Recent studies revealed that these antibodies have decreased in infected individuals with symptoms through time but decreased dramatically in asymptomatic individuals [68]. The S protein is the main target of antibodies and strongly induces immune cells to produce anti-SARS-CoV-2 IgG and IgA antibody [69, 70]. Neutralizing antibodies block the receptor-binding domain (RBD) of the viral spike protein from binding to its receptor ACE2 [39]. Unexpectedly, several studies reported that IgA antibody responses to the S protein occur earlier than IgM, and these findings are important for design of IgA-based serology tests [71, 72].

## 8. Escape Mechanisms of Coronaviruses from the Immune Responses

Current observations show that coronaviruses are exceptionally compatible with staying away from immune recognition and reducing immune reactions. This somehow elucidates why they tend to a long incubation period, 2 to 11 days [71]. Most mechanisms depend on the inhibiting innate immune responses, especially the detection and signaling of IFN-I. For MERS-CoV and SARS-CoV, the reaction to viral infection is suppressed by IFN-I [72]. Both coronaviruses use several strategies to intervene with the signaling leading to IFN-I generation and the interferon-*α* receptor (IFNAR) signaling downstream. The viral proteins include the nonstructural, and M proteins are the primary molecules in host immune modulation [73]. This damping approach is closely related to the severity of the disease. In the IFN-I induction phase, SARS-CoV interferes directly or indirectly with the downstream signaling of RNA sensors. For instance, ubiquitination and degradation of RNA sensor adaptor molecules mitochondrial antiviral signaling protein (MAVS), tumor necrosis factor receptor-associated factor (TRAF3/6), and IRF3 nuclear translocation inhibitors. MERS-CoV additionally uses some of these strategies with an extra mechanism, such as the histone modification suppressor [48]. When IFN-I is discharged, these two viruses are armed with a mechanism that inhibits IFN signaling. Reduction in STAT1 phosphorylation is a case in point [35].

In acquired immune escape, antigen presentation was downregulated through MHC class I and MHC class II, when the dendritic cells or macrophages became infected with MERS-CoV which would significantly reduce T cell activation [74]. Infection with MERS-CoV can prompt T cell apoptosis by activating both intrinsic and extrinsic apoptosis pathways in T cells which may lead to severe immunosuppression [47].

As an abovementioned, many anti-SARS-CoV-2 neutralizing antibodies detect RBD epitopes of the S protein and bind to this region, preventing the virus from binding to the ACE2 receptor [75–77]. Recent studies indicated that there are some mutations in the RBD epitope, and as a result, antibodies bound to them incompletely. Therefore, the virus escapes from humoral immunity [78, 79].

The cellular immune responses are suppressed with the expression of markers including programmed cell death protein 1 (PD1), T cell Ig, and mucin domain-3 protein (TIM-3) on T cells as well as secretion of anti-inflammatory cytokines such as IL-10 [80]. Studies on the expression of functional proteins on T cells have shown that although there was no significant difference in the expression of activation molecules of CD4^+^ T cells between the severe and mild group, the expression of T cell immunoglobulin, ITIM domain (TIGIT), and NKG2A on CD8^+^ T cells was considerably increased in the severe group [81, 82]. In brief, dysfunction of T cell subsets may ultimately reduce the host antiviral immunity; however, the exact mechanisms of suppression of the cellular immune response in COVID-19 patients have not yet been elucidated.

## 9. New Insights in the Design and Manufacture of Vaccines and Drugs for Immunotherapy against COVID-19 Infection

Numerous interventions claim that innate immunity performs a critical role in viral infections [72]. Some of these examples are IFN-I, antagonists of other proinflammatory cytokines, and antiviral agents. When utilizing IFN-I for treatment, the timing of administration in a mouse model of both SARS-CoV or MERS-CoV infection is critical to provide a protective response [83]. Also, the analysis of the immune protection and the long-term immune memory of convalescent persons may help to develop preventive and therapeutic measures for the subsequent outbreak of similar coronaviruses. Due to the quick increment of SARS-CoV-2 infections and affected countries, endeavors toward developing an effective SAR-CoV-2 vaccine have been initiated in several countries. The choice of target antigen and vaccine platform is likely found on MERS-CoV and SARS-CoV vaccine studies described in Table 1 to create the SAR-CoV-2 vaccine achievable. The collection of crucial data for vaccine development and assessment must be well described. This contains discovering objective antigen, injection route, connected-immune protection, animal models, versatility, processing facility, determination of outbreaks, and target society. Total S or S1, which contains RBD, could be considered as a proper vaccine antigen since it could instigate neutralizing antibodies that inhibit host cell binding and contamination. Although RBD is an interesting target for vaccines, studies have shown that its immunogenicity is weak. To overcome this limitation, some strategies such as using the dimer form of this protein as well as combining it with appropriate adjuvants have been suggested [84].

Table 1 describes the chosen antigens and platforms that have been tried for MERS-CoV and SARS-CoV in both preclinical and clinical investigations [7]. Recent developments of mRNA vaccines have enhanced the consistency and efficiency of protein translation; therefore, it could initiate effective immune responses [74, 85].

To date, numerous mutations in the amino acid protein COVID-19 especially in RBD have been identified, but their effects on binding to the host receptor are unknown [86]. China National Bioinformation Center examined the genetic diversity of different strains of COVID-19 and reported that 77,801 SARS-CoV-2 gene sequences were identified. Besides, the 15,018 mutations containing 14,824 mononucleotide polymorphisms have been observed [87]. Although candidate vaccines elicit antibody and T cell responses, it is not yet clear whether designed vaccines can prevent these mutations and protect vaccinated individuals. [88]. Moreover, the use of viral replication inhibitor drugs may cause mutations in the virus [89].

## 10. Conclusion

In summary, phagocytes, natural antibody, and cytokines have a key role in innate immunity against SARS-CoV-2 infection. The function of DC and macrophage was impaired in SARS-CoV infection; as a consequence, this virus is a weak inducer of IFN-I in the infected cell, unlike SARS-CoV-2. Also, the number of CD8^+^ T cells was increased in MERS and SARS-CoV, and it is related to the severity of diseases. CD4^+^ T cells and neutralizing antibodies were involved in SARS-CoV-2 infection and have a protective role in this disease. Therefore, due to the prominent role of the immune system in the defense against the COVID-19 virus, with the necessary and sufficient knowledge in this field (especially in the escape mechanisms of the virus from the immune system), the immune system manipulations can be used to prevent and treat this infection. The vaccine should be designed to elicit a humoral and cellular immune response to produce high-affinity neutralizing antibodies against SARS-CoV-2. For this purpose, the use of immunogenic RBD protected domains seems appropriate. However, for the treatment of diseases, compounds such as cytokines or antibodies act to strengthen the immune system against the virus can be used according to the patient's clinical condition.

## Figures and Tables

**Table 1:** Potential vaccine candidates developed for SARS-CoV and MERS-CoV.

Vaccine platform	Immunogen	Animal model	Route of injection	Adjuvant	Advantage	Disadvantage
Inactivated	Whole virus (i) Inactivated by formaldehyde or gamma irradiation	Mice	IM	Alum and CpG ODN	(i) Excellence in neutralizing Ab induction	(i) Possible cause hypersensitivity-type lung
					(ii) Can be combined with different adjuvant	(ii) Possible Th2-bias
						(iii) Cannot employ cell-mediated immunity
Live-attenuated virus	Mutant MERS-CoV and SARS-CoV	Mice, rhesus macaques	IN, IP	—	(i) Excellence in the induction of T and B cell responses	(i) Risk of relapse
					(ii) Site-directed mutagenesis can be tailor-made	(ii) Cold chain required
						(iii) Not suitable or sensitive population such as infants, immunocompromised, or elderly individuals
Subunit	Full-length spike, S1, RDB, nucleocapsid (i) Formulated with various adjuvants and/or fused with Fc	Mice, rhesus macaques	SC, IM, IN	MF59, Ontanide, Freund's adjuvant, alum, monophosphoryl lipid A, Montanide ISA51, CpG ODN	(i) High safety profile	(i) Need appropriate adjuvant
					(ii) Continuous production	(ii) Cost-effectiveness may change
					(iii) Induction of high titers of neutralizing antibodies	
					(iv) Local mucosal immune responses were observed in mice immunized through IN route	
DNA	Full-length spike or S1 (i) IM follow by electroporation	Mice, camels and rhesus macaques	IM	Without adjuvant	(i) Fast production	(i) Efficient delivery system required
					(ii) Simple design and manipulation	(ii) Induce lower immune responses when compared with a live vaccine
					(iii) Initiate both B and T cell responses	
Viral vector	Full-length spike or S1 (i) Vector used: ChAd or MVA	Mice, Bactrian camels	IM	CD40L	(i) Excellence in immune induction	(i) Different inoculation routes may produce different immune responses
						(ii) Possible TH2 bias
Virus-like particles	RDB, S, or coexpressing of S1, M, and E	Rhesus macaques, mice	IM	Alum, poly(I:C) CpG ODN	(i) Multimeric antigen display	(i) Need optimum assembly condition
					(ii) Maintain virus particle structure	

RBD: a receptor-binding domain in S1; MVA: modified vaccinia virus Ankara; ChAdOx1: chimpanzee adenovirus; IM: intramuscular; IN: intranasal; IP: intraperitoneal; SC: subcutaneous.

## Data Availability

The data used to support the findings of this study are available from the corresponding author upon request.
